# INFLUENCES ON CUSTODIAL GRANDCHILDREN'S RECEIVING TREATMENT FOR DISTRESS

**DOI:** 10.1093/geroni/igac059.2554

**Published:** 2022-12-20

**Authors:** Bert Hayslip, Julian Montoro-Rodriguez

**Affiliations:** University of North Texas, Denton, Texas, United States; University of North Carolina at Chatlotte, Charlotte, North Carolina, United States

## Abstract

Little work exists regarding the difficulties facing grandchildren raised by their grandparents, where previous research suggests such children to be especially at risk for psychological difficulties and that they often have limited access to potentially efficacious psychosocial interventions. The present study integrates these two perspectives in examining multiple sociodemographic, family-related, and grandchild-specific factors differentiating custodial grandchildren who were being treated for a variety of emotional and behavioral difficulties (N = 80) and those who were not (N = 157), as reported by the custodial grandparent. A MANOVA (F 23, 176 = 9.74, p < .01, eta2 = .56) indicated that custodial grandchildren who were receiving treatment came from larger grandfamilies, were older, were having a greater variety of grandparent reported psychosocial difficulties, and were experiencing more emotional, behavioral, attentional, and relationship problems. The grandparents of such grandchildren experienced more parental strain, reported less social support, were less resilient, were less satisfied with the grandparent role, and reported poorer health. They also reported being less strongly attached to the grandchild and expressed more negative affect toward him/her. However, they also reported more openness regarding a variety of difficulties that might be addressed by mental health professionals. A subsequent discriminant analysis reflecting a weighted linear combination of the above factors (X2 19 = 132.09, p < .01, Wilks’ Lambda = .498) correctly classified 86.1% of cases based upon treatment status. These findings provide a basis for understanding the determinants of custodial grandchilden’s receiving needed psychosocial interventions crucial to their well-being and adjustment.

